# Environment and Social Service for Aging in Place Decisions: A Different Scenario by Health Status

**DOI:** 10.1093/geroni/igaf122.1778

**Published:** 2025-12-31

**Authors:** Junmin Park, Yeonjung Lee

**Affiliations:** Chung-Ang University, Seoul, Republic of Korea; Chung-Ang University, Seoul, Republic of Korea

## Abstract

In the era of longer life spans, Aging in Place (AIP) has become a global topic. AIP goes beyond staying in the community and requires meeting various needs. A well-developed social service environment is key to effective AIP strategies, yet Korea still lacks sufficient support. This study examines the role of social services in Korea, considering the influence of the environment. Specifically, it examines how awareness of social services moderates the relationship between both physical and social environments and the planning for AIP, considering health status. Logistic regression was conducted using data from the 2023 National Survey of Older Koreans (n = 9,770). Those with higher satisfaction with both physical and social environment are more likely to AIP, when they are in good health. These relationships were stronger for people who have higher awareness of social services. However, when they are in deteriorating health, having higher satisfaction with physical environment was related to less intention to AIP, and these associations were only significant for older adults with more knowledge of social services. The results show that improving the suitability of physical and social environments for older people is essential. In addition to the need for diverse social services to support AIP regardless of health status, older adults must also be aware of the availability and accessibility of these services. Lastly, Korean older adults, who are generally reluctant to burden their children, require a tailored AIP strategy that emphasizes the need for a Respite Care system.

